# Eating Behavior and Childhood Overweight Among Population-Based Elementary Schoolchildren in Japan 

**DOI:** 10.3390/ijerph9041398

**Published:** 2012-04-16

**Authors:** Hirotaka Ochiai, Takako Shirasawa, Rimei Nishimura, Aya Morimoto, Naoki Shimada, Tadahiro Ohtsu, Masayasu Hashimoto, Hiromi Hoshino, Naoko Tajima, Akatsuki Kokaze

**Affiliations:** 1 Department of Public Health, Showa University School of Medicine, 1-5-8 Hatanodai, Shinagawa-ku, Tokyo 142-8555, Japan; Email: shirasawa@med.showa-u.ac.jp (T.S.); naokismd@med.showa-u.ac.jp (N.S.); tohtsu@med.showa-u.ac.jp (T.O.); koeizemi@med.showa-u.ac.jp (M.H.); hhiromi@med.showa-u.ac.jp (H.H.); akokaze@med.showa-u.ac.jp (A.K.); 2 Division of Diabetes, Metabolism and Endocrinology, Department of Internal Medicine, Jikei University School of Medicine, 3-25-8 Nishi-Shinbashi Minato-ku, Tokyo 105-8461, Japan; Email: rimei@jikei.ac.jp (R.N.); aya@jikei.ac.jp (A.M.); 3 Jikei University School of Medicine, 3-25-8 Nishi-Shinbashi Minato-ku, Tokyo 105-8461, Japan; Email: ntajima@jikei.ac.jp

**Keywords:** eating behavior, overweight, children, eating until full, chewing

## Abstract

This study investigated the relationship between eating behavior and childhood overweight among population-based elementary schoolchildren in Japan. Data was collected from fourth graders (9 or 10 years of age) from Ina Town, Saitama Prefecture, Japan from 1999 to 2009. Information about subjects’ sex, age, and lifestyle, including eating behaviors (eating until full and chewing thoroughly), was obtained using a self-administered questionnaire, and height and weight were measured directly. Overweight was determined according to the definition established by the International Obesity Task Force. Data from 4027 subjects (2079 boys and 1948 girls) were analyzed. Chewing thoroughly was associated with a significantly decreased odds ratio (OR) for being overweight, whereas eating until full significantly increased the OR for being overweight (OR: 1.50, 95% confidence interval: 1.16–1.94) among boys. However, eating until full was not associated with a significantly increased OR for being overweight among the group that reported chewing thoroughly, whereas it was associated with a significantly increased OR for being overweight (2.02, 1.38–2.94) among boys who did not chew thoroughly. In conclusion, eating until full or not chewing thoroughly was associated with being overweight among elementary schoolchildren. Results of this study suggest that chewing thoroughly may be an avenue to explore childhood overweight prevention efforts.

## 1. Introduction

Obesity plays a central role in insulin resistance and metabolic syndrome, which includes hyperinsulinemia, hypertension, hyperlipidemia, and type 2 diabetes mellitus [1]. A recent study reported that pediatric metabolic syndrome was a significant predictor of metabolic syndrome and type 2 diabetes mellitus in adulthood [2]. Moreover, obesity in childhood was strongly associated with increased rates of premature death [3]. Thus, prevention of childhood obesity is of great importance.

Adolescence is a critical period for the development of obesity [4,5]. Obesity that begins at the period appears to increase the risk of persistent obesity and its complications [5]. Since strong associations between lifestyle factors and obesity have been reported [6,7,8,9,10], it is very important to improve lifestyles before adolescence, which could contribute to the prevention of overweight/obesity among adolescents and adults. 

Among lifestyle factors, eating behavior has been shown to be a factor related to body mass index (BMI), overweight, and obesity [11,12,13,14,15,16]. For example, eating until full (eating a large quantity of food in one meal, unrelated to eating disorders [11]) was associated with being overweight [11,12] among adults, whereas chewing or not chewing frequently was also reported to be related to BMI or obesity [13,14,15,16] among children and adults. However, to the best of our knowledge, there are no studies about the association between eating until full and being overweight among population-based elementary schoolchildren. Furthermore, the impact of eating until full on overweight could differ based on whether subjects chew thoroughly or not, because the total energy intake among those who do not chew thoroughly and then eat until full could be higher than among those who chew thoroughly and then eat until full. Therefore, when eating until full is evaluated as a factor associated with being overweight or obese, it may be essential to consider chewing.

Accordingly, the present study was conducted to investigate the relationship between eating behavior (eating until full and chewing) and being overweight among population-based elementary schoolchildren in Japan.

## 2. Methods

The town of Ina is located in Saitama Prefecture, Japan. In addition to the annual national health checkups performed in accordance with the School Health Law of Japan, the town has been conducting a unique health-promotion program since 1994. The program consists of a self-administered questionnaire survey and blood and physical examinations for fourth and seventh graders; several studies related to this program have been published [17,18,19,20]. The present study was conducted as part of the program. 

### 2.1. Study Subjects

The study population was drawn from the 4106 fourth-grade schoolchildren (9 or 10 years of age) from Ina during 1999–2009. Of these, 25 refused to participate in the program (participation rate: 99.4%). We excluded 54 participants in the analysis because of missing data about the subject’s height, weight, and eating behaviors (eating until full and chewing thoroughly). Thus, data from 4027 subjects (2079 boys and 1948 girls) were analyzed. Informed consent prior to participation in the study was obtained from the parent or guardian of each subject. The study protocol was approved by the Medical Ethics Committee of Showa University School of Medicine.

### 2.2. Questionnaire Survey

A self-administered questionnaire was distributed to each subject by the school teacher in the elementary school. Then, each subject and the parent or guardian completed the questionnaire. The questionnaire consisted of two sections: one section (front side) designed to be completed by the subject and the other section (back side) designed to be completed by the parent or guardian. 

The following information was collected using the questionnaire completed by each subject: sex, age, exercise other than physical education class (daily, sometimes, or none), snacking after dinner (always, often, seldom, or none), eating until full (yes or no), and chewing thoroughly (yes or no). The information regarding chewing thoroughly was obtained from the question “Do you chew thoroughly when you eat?” 

Each subject’s parent or guardian was asked to complete the questionnaire regarding the subject’s birth weight, wake-up time, bedtime, frequency of eating breakfast (daily, sometimes, or none), and family information (whether the child was an only child, and the parents’ heights and weights). Sleep duration was calculated from wake-up time and bedtime. Frequency of eating breakfast was categorized into two groups: skipping breakfast (sometimes and none) and not skipping breakfast (daily).

### 2.3. Anthropometric Measurements

The height and weight of each subject were measured in the school’s infirmary or in a designated room to protect privacy during the procedures. For anthropometric measurements, subjects wore light clothing but no shoes and socks. Height was measured to the nearest 0.1 cm using a stadiometer (TK-11253, Takei Scientific Instruments Co., Ltd., Japan), and body weight was measured to the nearest 0.1 kg using a scale (TBF-102, Tanita, Japan). BMI was calculated as weight (kg) divided by height (m) squared. These measurements were recorded annually from 1999 to 2009.

### 2.4. Definition of Overweight

Childhood overweight (including obesity) was determined according to the age- and sex-specific cut-off points proposed by the International Obesity Task Force [21]. Overweight (including obesity) in parents was defined as a BMI ≥ 25 kg/m^2^, according to the World Health Organization criteria [22]. 

### 2.5. Data Analysis

The Chi-squared test and the Wilcoxon rank-sum test were used to compare characteristics between the overweight and non-overweight groups. To evaluate the relationship between eating behavior and being overweight, a logistic regression model was used. We first estimated the crude odds ratio (OR) for being overweight and 95% confidence interval (95% CI), and then adjusted for potential confounders. Variables that have been reported to be related to being overweight [6,10,23,24,25,26] and that were different between the overweight and the non-overweight groups with a *P* value < 0.05 were considered potential confounders. All statistical analyses were performed using Statistical Analysis System (version 9.2). A *P* value < 0.05 was considered statistically significant for all analyses.

## 3. Results

Some baseline characteristics (birth weight, exercise, chewing thoroughly, BMI, proportion of overweight) were significantly different between boys and girls (*P *< 0.05). Therefore, we analyzed the data by each sex. The characteristics of overweight and non-overweight study participants (boys) are shown in Table 1. Children with one or both parents who were overweight and children with no siblings were more frequently found in the overweight group. Statistically significant differences between the overweight and non-overweight groups were observed for birth weight and exercise. Those who reported eating until full were more frequently found in the overweight group. The proportion of those who reported chewing thoroughly in the overweight group was significantly lower than that in the non-overweight group.

**Table 1:** Characteristics of non-overweight and overweight study participants (boys).

Variables			Number of		Non-overweight		Overweight		*P* value ^a^
			missing data		(n = 1701)		(n = 378)		
Age (years)			0		9.0 (9.36)		9.0 (9.39)		0.363
								
Birth weight (g) (%)			0						
	<2500				10.2		7.7		<0.001
	2500–2999				30.0		26.2		
	3000–3499				43.7		41.3		
	3500–3999				14.6		21.2		
	4000+				1.5		3.7		
Overweight parent (%)			0						
	None				72.7		49.7		<0.001
	Father only				19.6		28.0		
	Mother only				5.4		13.2		
	Father and mother				2.4		9.0		
								
No siblings (%)			11		9.9		15.4		0.002
								
Sleeping hours (%)			98						
	10.0+				16.6		11.7		0.082
	9.0–9.9				61.0		62.1		
	8.0–8.9				20.4		23.4		
	<8.0				2.0		2.8		
								
Exercise (%)			48						
	None				13.7		17.1		<0.001
	Sometimes				23.1		30.7		
	Daily				63.3		52.2		
								
Skipping breakfast (%)			15		3.1		3.2		0.959
								
Snacking after dinner (%)			9						
	Seldom or none				50.2		48.7		0.584
	Always or often				49.8		51.3		
								
Eating until full (%)			0		63.7		70.9		0.008
								
Chewing thoroughly (%)			0		67.5		42.9		<0.001

Except for percentages (%), values are medians (means). ^a^ Chi-squared test or Wilcoxon rank-sum test.

Table 2 shows the characteristics of overweight and non-overweight study participants (girls). Girls with one or both parents who were overweight were more frequently found in the overweight group. Statistically significant differences between the overweight and non-overweight groups were observed for birth weight, sleeping hours, exercise, and snacking after dinner. There was no significant difference found between the overweight and non-overweight groups in eating until full, whereas the proportion of those who reported chewing thoroughly in the overweight group was significantly lower than that in the non-overweight group.

**Table 2:** Characteristics of non-overweight and overweight participants (girls).

Variables			Number of		Non-overweight		Overweight		*P* value ^a^
			missing data		(n = 1674)		(n = 274)		
Age (years)			0		9.0 (9.36)		9.0 (9.35)		0.738
								
Birth weight (g) (%)			0						
	<2500				11.6		9.1		0.002
	2500–2999				37.4		29.6		
	3000–3499				40.0		46.4		
	3500–3999				10.3		12.4		
	4000+				0.7		2.6		
								
Overweight parent (%)			0						
	None				71.5		52.6		<0.001
	Father only				20.3		27.0		
	Mother only				5.8		9.1		
	Father and mother				2.5		11.3		
								
No siblings (%)			24		9.7		12.9		0.109
								
Sleeping hours (%)			91						
	10.0+				13.8		15.4		0.045
	9.0–9.9				60.6		51.7		
	8.0–8.9				23.3		29.3		
	<8.0				2.3		3.5		
								
Exercise (%)			46						
	None				26.7		34.2		0.013
	Sometimes				31.8		32.7		
	Daily				41.5		33.1		
								
Skipping breakfast (%)			17		4.0		2.2		0.142
								
Snacking after dinner (%)			14						
	Seldom or none				52.7		43.5		0.005
	Always or often				47.3		56.5		
								
Eating until full (%)			0		64.0		62.8		0.686
								
Chewing thoroughly (%)			0		83.4		67.5		<0.001

Except for percentages (%), values are medians (means). ^a^ Chi-squared test or Wilcoxon rank-sum test.

Table 3 shows crude and adjusted ORs of eating behavior (eating until full or chewing thoroughly) for being overweight. Among boys, eating until full significantly increased the OR for being overweight (OR: 1.50, 95% CI: 1.16–1.94), while chewing thoroughly was associated with a significantly decreased the OR for being overweight (0.37, 0.29–0.46). In girls, chewing thoroughly significantly decreased the OR for being overweight (0.45, 0.33–0.62), whereas the adjusted OR of eating until full was not statistically significant. The interaction of sex and eating until full on being overweight was statistically significant (*P *= 0.036).

**Table 3:** Crude and adjusted odds ratios of eating until full or chewing thoroughly for being overweight.

Variables				Total		Overweight		Crude			Adjusted	
				N		n (%)		OR	95% CI		OR	95% CI
Among boys												
	Eating until full											
		Yes		1351		268 (19.8)		1.39	1.09–1.77		1.50	1.16–1.94
		No		728		110 (15.1)		1.00			1.00	
											
	Chewing thoroughly											
		Yes		1310		162 (12.4)		0.36	0.29–0.45		0.37	0.29–0.46
		No		769		216 (28.1)		1.00			1.00	
										
Among girls												
	Eating until full											
		Yes		1244		172 (13.8)		0.95	0.73–1.23		0.96	0.72–1.27
		No		704		102 (14.5)		1.00			1.00	
											
	Chewing thoroughly											
		Yes		1581		185 (11.7)		0.41	0.31–0.55		0.45	0.33–0.62
		No		367		89 (24.3)		1.00			1.00	

OR, odds ratio; CI, confidence interval. Adjusted for birth weight, overweight parent, no siblings, and exercise among boys. Adjusted for birth weight, overweight parent, sleeping hours, exercise, and snacking after dinner among girls.

Next, the adjusted ORs of eating until full for being overweight were calculated separately for the chewing thoroughly or not groups (Table 4). When the analysis was performed for boys, eating until full significantly increased the OR (2.02, 1.38–2.94) for being overweight among the group that did not chew thoroughly. However, it did not significantly increased the OR among the group that did chew thoroughly. When the analysis was limited to girls, a significantly increased OR was not observed regardless of chewing thoroughly or not. The combination of the two eating behaviors had the interaction on being overweight among boys (*P *< 0.001).

**Table 4:** Adjusted odds ratios of eating until full for being overweight by chewing thoroughly or not.

				Chewing thoroughly						Not chewing thoroughly				
				Total		Overweight		AOR (95% CI)		Total		Overweight		AOR (95% CI)
				N		n (%)				N		n (%)		
Among boys														
	Eating until full													
		Yes		841		103 (12.3)		1.10 (0.77–1.57)		510		165 (32.4)		2.02 (1.38–2.94)
		No		469		59 (12.6)		1.00		259		51 (19.7)		1.00
												
Among girls														
	Eating until full													
		Yes		1002		110 (11.0)		0.88 (0.63–1.23)		242		62 (25.6)		1.08 (0.61–1.92)
		No		579		75 (13.0)		1.00		125		27 (21.6)		1.00

AOR, adjusted odds ratio; CI, confidence interval. Adjusted for birth weight, overweight parent, no siblings, and exercise among boys. Adjusted for birth weight, overweight parent, sleeping hours, exercise, and snacking after dinner among girls.

The adjusted ORs of combinations of chewing thoroughly and eating until full for being overweight were shown in Table 5. Compared to the combination of chewing thoroughly and not eating until full, “not chewing thoroughly and eating until full” and “not chewing thoroughly and not eating until full” significantly increased the OR for being overweight. Furthermore, the increase in OR of “not chewing thoroughly and eating until full” was more pronounced than that of “not chewing thoroughly and not eating until full”. “Chewing thoroughly and eating until full” didn’t significantly increase the OR among each sex. 

**Table 5:** Adjusted odds ratios of combinations of chewing thoroughly and eating until full for being overweight.

		Boys				Girls		
		Overweight		AOR (95% CI)		Overweight		AOR (95% CI)
		n/N (%)				n/N (%)		
Chewing thoroughly and not eating until full		59/469 (12.6)		1.00		75/579 (13.0)		1.00
Chewing thoroughly and eating until full		103/841 (12.3)		1.09 (0.76–1.57)		110/1002 (11.0)		0.88 (0.63–1.23)
Not chewing thoroughly and not eating until full		51/259 (19.7)		1.79 (1.17–2.77)		27/125 (21.6)		1.94 (1.14–3.30)
Not chewing thoroughly and eating until full		165/510 (32.4)		3.58 (2.52–5.08)		62/242 (25.6)		2.09 (1.39–3.15)

AOR, adjusted odds ratio; CI, confidence interval. Adjusted for birth weight, overweight parent, no siblings, and exercise among boys. Adjusted for birth weight, overweight parent, sleeping hours, exercise, and snacking after dinner among girls.

## 4. Discussion

To the best of our knowledge, this is the first study concerning the relationship between eating behavior, specifically eating until full and chewing, and being overweight among population-based elementary schoolchildren. In this study, chewing thoroughly decreased the OR for being overweight for both sexes, while eating until full increased the OR for being overweight among boys. Furthermore, eating until full did not increase the OR among those who chewed thoroughly, but it increased the OR among boys who reported not chewing thoroughly.

In the present study, some baseline characteristics such as birth weight, parental overweight, no sibling, and lifestyle factors (sleeping hours, exercise, and snacking) were significantly different between the overweight and non-overweight groups. These factors have been shown to be associated with childhood overweight or obesity [10,24,25,26,27,28]. Therefore, we adjusted these factors in the analysis to evaluate the relationship between eating behavior (eating until full and chewing thoroughly) and childhood overweight. As a result, eating until full or not chewing thoroughly was significantly associated with being overweight, independent of these factors.

A previous study showed that “not chewing frequently” significantly increased the OR for obesity among preschool children [14]. In addition, obesity has been reported to be associated with less time chewing [15,16]. These results are consistent with our study results. Stimulation induced by mastication has been shown to affect satiety by increasing histamine release from the satiety control center of the brain [29]; this suggests that chewing thoroughly helps protect against eating too much. Furthermore, a recent study suggested that chewing thoroughly decreases the rate of eating, and eating slowly may help maximize satiation, resulting in reducing energy intake during meals [30]. In fact, linear eaters—that is, people who eat at an approximately constant rate—ate less food when challenged to eat at a lower rate [31]. Therefore, chewing thoroughly could be a effective tool for the prevention of overeating and becoming overweight during childhood.

As shown in Table 3, the adjusted OR of eating until full for being overweight was significantly increased in boys, which was consistent with results of recent studies [11,12]. The reason could involve differences in energy intake between those who eat until full and those who do not. Maruyama *et al*. showed that people who reported eating until full had higher total energy intake than those who did not report eating until full [11]. Therefore, eating until full could lead to higher energy intake, and contribute to the OR seen in Japanese schoolchildren in this study.

In the present study, the impact of eating until full on being overweight differed if subjects chewed thoroughly or not; eating until full was associated with a significantly increased OR for being overweight among boys who did not chew thoroughly, whereas it was not associated with an increased OR for being overweight among those who chewed thoroughly. These results suggest that eating until full is not a risk factor for being overweight if people chewed thoroughly. A previous study showed that people who take many bites over a given time period consume less food in grams and less energy per time period than those who take few but big bites [32]. Additionally, chewing thoroughly was reported to help maximize satiation [30]. Therefore, the amount of total energy intake among those who chew thoroughly and eat until full could be smaller than that among those who do not chew thoroughly and eat until full. Actually, chewing thoroughly significantly decreased the ORs for being overweight among each sex regardless of eating until full or not in our study; the ORs of chewing thoroughly were 0.31 among boys who ate until full and 0.45 among girls who ate until full, while they were 0.56 among boys who didn't eat until full and 0.51 among girls who didn't eat until full. Accordingly, the combination of chewing thoroughly and eating until full may not lead to excess energy intake, resulting in no substantial impact on overweight status. Because information on total energy intake was not collected in our study, further study will be needed to verify these study results.

In our study, “not chewing thoroughly and eating until full” and “not chewing thoroughly and not eating until full” significantly increased the OR for being overweight, compared to “chewing thoroughly and not eating until full”. However, “chewing thoroughly and eating until full” did not significantly increase the OR. These results suggest that “not chewing thoroughly” may be a risk factor for being overweight. Therefore, chewing thoroughly could be an effective intervention target among elementary schoolchildren.

The strength of this study is the direct measurement of height and weight as well as the fact that questionnaires were administered to more than 4000 population-based schoolchildren. We are the first group, to our knowledge, which evaluated the relationship between eating behaviors (chewing thoroughly and eating until full) and being overweight among elementary schoolchildren. However, potential limitations should be discussed. First, information about eating until full and chewing thoroughly in this study may be not objective because it was self-reported. Therefore, it would have been better if we could have used a form of objective measurement to estimate eating behaviors, such as laboratory-based methods [32]. However, it is obviously difficult to apply such methods to a large epidemiological study such as this study. Second, the validation of the questionnaire used in our study was not confirmed. Therefore, to verify our study results, a validated questionnaire should be used in future research. Finally, the present study could not determine the causal relationship between eating behavior and overweight because of its cross-sectional design. Therefore, the possibility of reverse causality cannot be denied.

## 5. Conclusions

Eating until full or not chewing thoroughly was associated with being overweight among elementary schoolchildren. However, eating until full might not be related to being overweight if subjects chewed thoroughly. The results of this study suggest that chewing thoroughly may be an effective strategy in helping children to avoid becoming overweight. Accordingly, a public health campaign aimed at improving eating behavior might help decrease the risk of becoming overweight among schoolchildren. Since health education is already included in the education at elementary schools in Japan, eating behavior, especially chewing thoroughly, should be emphasized in the health education of all elementary schools.
